# Typhoid Fever in Animals*From the transactions Pathological Society of London.

**Published:** 1886-04

**Authors:** J. Bland Sutton


### Art. XI.—TYPHOID FEVER IN ANIMALS.*

BY J. BLAND SUTTON, M. D.

The question as to the existence of typhoid fever in animals other than man is of some interest, and it is one concerning which we know very little. Dr. Budd, among some of the pa-

* From the transactions Pathological Society of London.

pers he wrote in connection with the subject of comparative pathology, put forward the opinion that the "acute infective fever" of the pig known as "swine fever, pig typhoid, purples, or red soldier," was really the same disease which we recognize as typhoid fever in man.

The symptoms, etiology, and morbid anatomy of this disease of the pig are carefully and ably set forth in a very interesting paper in the *Veterinarian* for 1875, by Professor Wortley Axe, who also tries to prove the identity of pig and human typhoid fever.

Dr. Klein in the *Path. Soc. Trans.*, vol. xxviii, details an account of the morbid anatomy of the disease as seen in the pig, and shows, most conclusively, that the disease in this animal is totally different from that seen in man. One of the chief points in the argument is, that in the pig the ulcerated patches in the bowel, or the nodules, have nothing whatever to do with lymphatic follicles, whereas in man it is these structures which are affected.

It should be mentioned that some observers hold the view that the so-called pig typhoid is anthrax, but Dr. Klein's experiments clearly negative this opinion. Seeing then that swine fever and human typhoid are not identical, the question becomes still more pressing. Do animals suffer from typhoid? The following cases have some bearing on this matter. In 1882, while making a *post-mortem* examination on a lemur which had died in the Zoological Gardens, from perforation of the ileum near the cæcum, the Peyer's patches were found to be ulcerated in the same manner, and presented the typical appearance as these structures do under the same condition in man. No other organs presented lesions of note. For some days before death, the lemur had suffered from profuse diarrhœa, the keeper experiencing considerable difficulty in keeping the cage clean. I was so positive that the ulcerations were typhoid that the deaths of other monkeys were predicted. In company with the late Mr. W. A. Forbes, who was at that time Prosector to the Zoological Society, an attempt was made to take clinical observations; this was impossible, and respect for the integrity of our skins made us relinquish the endeavor.

Seven days after, the lemur which lived with the first patient died, having suffered for many days with diarrhœa and bloody stools. Death was occasioned by very profuse hæmorrhage. At the *post-mortem* examination, the Peyerian patches were found ulcerated from the commencement of the ileum, throughout the whole of the large intestine to the point where the ridge of adenoid tissue marks the line of fusion of rectum and anus, (hind-gut and the proctodæum of embryology). I have since found that this part of the rectum is liable to become ulcerated in man in severe cases of typhoid fever. About the same time, two monkeys died with exactly similar lesions, but confined to the ileum, also a tiger. On making sections of the Peyer's patches in the case of the tiger, and comparing them with sections taken from the human subject at about corresponding dates of the disease, the details coincided in a very marked manner.

At the time these cases occurred at the Zoological Gardens, typhoid fever was raging in the neighboring district and Camden Town.

With regard to the distribution of the lesions in the lemurs, some further facts are necessary. The ulcers extended from the ileum throughout the whole length of the large intestine, and were as large in the colon as in the ileum. This was a rather puzzling circumstance, for it had long been believed by comparative anatomists that the solitary glands were scattered in an irregular manner throughout the alimentary canal, from duodenum to rectum, whilst the agminate glands of Peyer were confined to the small intestine, being most numerous in the lower part of the ileum.

Such is not the case, for Mr. F. E. Beddard in a paper on "Hapalemur griseus," * one of the Lemuroids, records the existence of Peyer's patches in the cæcum, and in the colon to near its termination. In this mammal, the small intestine measured two feet, four inches in length and the large intestine one foot. The cæcum contained two agminate patches and the colon ten, in addition to the ordinary agminate glands in the small intestine.

In the July number of the *Journal of Anatomy and Physiology* vol. xviii, 1884, Dr. Dobson records the existence of Peyer's patches (agminate glands), in the cæcum and colon of certain insectivorous mammals and rodents. In some Insectivora,

---

* *Proc. Zoo. Soc.*, part iii, 1884.

such as Chrysochlriodæ and Talpidæ, the agminate glands were found to occur at intervals throughout the whole intestinal tract from the duodenum to the rectum.

Mr. Beddard has found, since the date of publication of the paper on Hapalemur, that a similar distribution of Peyer's patches is common among the Lemuroids, he having observed them in the large intestine of lemur catta, galago, and the slow loris. These interesting observations settle the question of peculiarity relative to the distribution of the lesions.

After diligent search I came across some observations, similar to my own, regarding typhoid fever in monkeys. Rayer in an article on "Fièvre Typhoide chez les Animaux," contained in the *Archives de Médecine Comparée*, 1843, mentions that during the months of November and December, 1839, typhoid fever broke out among the monkeys in the ménagerie of the Muséum d'Histoire Naturelle, Paris. On this occasion, M. Serres, who had previously observed the affection in monkeys, dogs, and cats, and had made careful preparations of the intestinal lesions, was able to make careful observations on the animals during life. The symptoms were very striking, being diarrhœa, increased frequency of pulse, and fever ending almost always in death. These observations of M. Serres and my own are, I believe, the only facts recorded regarding this disease in quadrumana.* No further opportunity for studying this affection presented itself until January in the present year (1885). The Zoological Society received, on deposit, six Canadian beavers apparently in the best of health. These animals had sojourned for a time at Liverpool previously to their transmission to London.

Not long after their arrival one of them died, and on making a *post-mortem* examination, I as surprised to find that the follicles of the intestines from the duodenum to the middle of the colon were ulcerated; the Peyerian patches from the middle of the jejunum to their termination at the ileo-cæcal valve were occupied with sloughs, the limits of which were determined by the size of the affected gland. In some of the patches the

* A case illustrating this question occurred recently in the Central Park Menagerie. A monkey, which had suffered from what was supposed to be simple diarrhœa with hemorrhage from the bowels, died, and a *post mortem* examination revealed extensive ulcerations of Peyer's Patches.—Eds.

edges were slightly rounded and indurated. The majority of the ulcers had the muscular coat for their base. On making microscopic sections through the affected patches, no structure could be distinguished. The mesenteric glands were enlarged.

The conclusion was that the case was one of typhoid fever, and that in all probability the five remaining beavers would die also. In this I was not disappointed; at least three of them came into my hands in the course of the next four or five weeks. At the end of that time it was deemed expedient to return the surviving two to their owner. Some two months later I learned that these two animals had recovered and were in excellent condition. The intestines in the other cases presented identical appearances. In one case the ulcers had penetrated beyond the muscular coat, and had given rise to intense peritonitis with abundant fœtid purulent fluid in the peritoneal cavity, but no perforation could be detected even on allowing a current of water to pass through the unopened bowel.

In two of the beavers the liver was the seat of some curious changes. The cut surface presented to the naked eyes numerous whitish specks just visible. On making sections through them the liver-cells presented a cloudy condition, and the hepatic cells in the immediate neighborhood of the abnormal specks presented a disintegrated appearance. Such phenomena are occasionally seen in human subjects who have died from this disease.

It is necessary to show that the disease is not tubercular. In the first place, tubercular ulceration of the bowels is, in animals, an exceedingly rare affection, indeed so rare that I have only once seen it, and that was in a coatimundi from South America, the subject of general tuberculosis. In this case, the appearance of the lesions was totally different, presenting a thickened nodular mass rising above the general level of the bowel, the peritoneum being involved in the thickening, and, what is still more significant, the tubercle-bacillus was present in enormous numbers; whereas, in the case of the beavers no tubercle-bacillus could be discovered, even after the most careful examination by myself or my friend Mr. Hudson, who quite independently submitted some of the sections to bacilli reagents.

In the beavers, the young ones were the first to succumb, and in them the intestinal lesions were the most marked. The disease extended over a period of six weeks altogether, the most obvious symptoms being disinclination for food and profuse diarrhœa.

The ulcers could be traced in the different parts of the small bowel, through the various stages from the normal condition to infiltration and sloughing, and in many parts the sloughing portion still sticking to the gut exhibited the peculiar bile-stained appearance so often seen in the human subject.

Lastly, I know of no other condition of the bowel, besides tuberculosis, likely to be confounded with them, and feel perfectly convinced in my own mind that we have here to deal with genuine typhoid lesions.

There have been many attempts on the part of veterinarians, particularly the French, to prove that the horse is liable to typhoid fever, but the diagnosis has been chiefly based on clinical grounds, and curiously enough, in outbreaks of what the French called typhoid fever in the horse the rate of mortality is so low as not to afford any opportunities of *post-mortem* investigation. I have not had an opportunity of studying the pathological lesions in either horse or ass. During the long vacation I made a special visit to the museum of the Veterinary School at Alfort. Some specimens of ulcerated intestine are there preserved, which present lesions exactly like those of human typhoid, but more particulars are necessary before one could decide with certainty that horses suffer from this affection. Coming back to the specimens concerned in this communication, I think it may be fairly claimed that they are really typhoid lesions, and that, with the solitary exception of M. Serres, they are the most authentic cases of typhoid fever yet recorded in animals. They possess a certain amount of value, for it is usually regarded impossible to inoculate an animal, other than man, with the typhoid poison. However, these cases prove that animals may acquire the disease, and are of tenfold more value than all the inoculation experiment that have yet been carried out on the transmission of this disease to animals, for up to the present time they have all been negative.

